# Patient satisfaction from ENT telephone consultations during the coronavirus disease 2019 pandemic

**DOI:** 10.1017/S0022215120002480

**Published:** 2020-11-17

**Authors:** M Zammit, R Siau, C Williams, A Hussein

**Affiliations:** 1 ENT Department, Broadgreen Hospital, Liverpool, UK; 2 ENT Department, Liverpool University Hospitals, UK; 3 ENT Department, Ain Shams University, Cairo, Egypt

**Keywords:** Telehealth, Telephone, Remote Consultation, Patient Satisfaction, Coronavirus

## Abstract

**Background:**

Telephone consultations have rapidly increased in the out-patient setting because of the coronavirus pandemic. A quality improvement project was implemented to improve patient satisfaction of telephone consultations in our unit.

**Methods:**

This was a prospective complete-cycle project. Patient satisfaction questionnaires were sent to patients following telephone consultations in ENT clinics. Based on a literature review and initial results, clinicians were encouraged to follow a structured consultation format. A second questionnaire survey was conducted following its implementation.

**Results:**

One hundred patient questionnaires were collected during the survey (April and June 2020). There was significant improvement over the two surveys in terms of satisfaction scores (*p* = 0.026), along with a significantly increased preference for telephone consultations over face-to-face consultations (*p* = 0.021).

**Conclusion:**

This study showed significant improvement in patient satisfaction and an increased telephone consultation preference through the use of a structured consultation model. The potential benefits in terms of infection control and impact on out-patient workload may see telephone consultations persist in the post-coronavirus era.

## Introduction

The recent coronavirus outbreak has presented a number of challenging circumstances for the healthcare system.^1^ Chief concerns revolve around treating and contact tracing coronavirus disease 2019 (Covid-19) patients and carriers, whilst maintaining adequate levels of elective and emergency patient care, and keeping the welfare of healthcare professionals in mind.^2^

The use of telephone consultations in place of traditional face-to-face consultations, where appropriate, has been instrumental in maintaining elective out-patient activity during the Covid-19 pandemic. This adjustment has also reduced patient footfall in hospital settings and assisted with social distancing in out-patient waiting areas.^1,3–5^

Telephone consultation facilities have also helped create a ‘triage system’, filtering vulnerable patients with stable conditions whilst identifying those who require necessary reviews for acute and urgent oncological conditions.^3^ However, this rapid change in practice has presented many clinicians with a steep learning curve, as generally telephone consultations were not widely used in ENT clinics in the UK during the pre-coronavirus era.

Meanwhile, Covid-19 has not relented, inferring an indefinite extension of telephone consultation use for the foreseeable future.^1^ In response to these challenges on clinical care, we have devised a quality improvement project to assess patient satisfaction and reception of telephone consultations in our unit, using feedback obtained to address any deficiencies outlined. We propose the use of a structured telephone consultation model to standardise telephone consultations, with the scope of improving communication and patient satisfaction.

## Materials and methods

A complete-cycle quality improvement project was undertaken. Patients undergoing telephone consultations in ENT clinics (head and neck, rhinology, otology, and balance clinics) were included. Telephone consultations were undertaken by five consultants – three senior registrars and two audiologists – for both surveys (described below). Patients deemed unsuitable for a telephone consultation included those requiring urgent face-to-face review (such as emergencies, and those with a high suspicion of malignancy or symptoms refractory to treatment) and patients without access to a confidential telephone line.

An initial survey was performed using an online uniform resource locator (‘URL’) address directing patients to an online questionnaire. Based on these results and a current literature review, a two-pronged intervention was designed, consisting of staff education and the application of a model structured telephone consultation framework. A follow-up survey was subsequently undertaken using the same online questionnaire completed by a second cohort of patients after undergoing telephone consultation in the ENT clinic. The first survey was conducted from 23rd March 2020 to 10th April 2020, with the second survey conducted from 8th June 2020 to 30th June 2020.

Throughout both time periods, the numbers of telephone consultations performed by the department for new referrals and for follow-up appointments were collected.

### Questionnaire contents

The questionnaire (Appendix 1) was created using Google Sheets. It consists of 26 questions, including the 21-point Medical Interview Satisfaction Scale (‘MISS-21’), widely used to assess patient satisfaction.^4^ Formulated by Meakin and Weinman, the Medical Interview Satisfaction Scale consists of 21 questions covering 4 areas of patient satisfaction: distress relief, communication comfort, rapport and compliance intent. Each question was rated by patients on a seven-point Likert scale (score of 1 = strongly disagree, 7 = strongly agree), with a total possible score of 147. When calculating scores for the communication comfort subscale, ratings were inverted (i.e. score 1 = 7, 2 = 6, etc.) to represent replies for ‘double negative’ answers.

The questionnaire also contained an overall rating (out of five) for the telephone consultation. Participants’ preferences for telephone consultations versus face-to-face appointments were also rated. A final field welcoming any additional comments regarding the telephone consultation was included at the end of the questionnaire.

### Statistical analysis

The Mann–Whitney U test was used for statistical comparisons between questionnaire ratings of the two survey cohorts, whilst patient preference between cohorts was compared using the chi-square test. The difference in number of follow-up consultations between the two time periods was also analysed using the chi-square test.

A *p*-value of less than 0.05 was considered to be statistically significant (95 per cent confidence interval). Statistical tests were performed using SPSS® software, version 23.

### Implemented change

Prior to initiating a second survey, results of the first survey were reviewed and discussed in a departmental meeting. The *BMJ* have published ‘information for practice’ regarding telephone consultations, together with guidance on telephone consultation during the Covid-19 pandemic; these were reviewed during the meeting.^5–7^ A structured framework for future telephone consultations, adapted from Marshall *et al*.,^8^ was agreed upon and implemented immediately (Table 1). A physical print-out of this model was placed in every ENT out-patient clinic room.

**Table 1.** Telephone consultation structured approach

Set-up	Have relevant guidelines, medical records & investigations to hand
Introduction	Introduce self: name, position, location Identify patient's name, date of birth & location
Situation	Explain reason for calling Ensure patient is happy with their environment Use pre-emptive phrases or ‘warning shots’ when delivering bad news* If call is urgent, say so
Background (active listening: picking up verbal cues)	Obtain clinical history & elicited symptoms Address ideas, concerns & expectations
Assessment	Explain any necessary investigations or results Any Covid-19 symptoms? Self-isolating or high-risk patient?
Review (opportunity for patients’ questions)	Ideas, concerns, expectations been met? Any further questions? Telephone or face-to-face follow up required?

* For example, ‘I'm sorry, I have some bad news’. Covid-19 = coronavirus disease 2019

## Results

### Cohort characteristics

In the first survey (23rd March to 10th April 2020), 138 patients had a telephone consultation; 48 of these patients (34.8 per cent) returned their questionnaires. In the second survey (8th–30th June 2020), 180 patients underwent telephone consultation; 52 of these patients (29 per cent) returned completed questionnaires. Patients’ characteristics are shown in Table 2.

**Table 2.** Patient demographics

Characteristic	1st survey	2nd survey
Gender ratio (F:M)	1.2:1	1.6:1
Age distributions (*n*)		
– 18–29 years	3	7
– 30–49 years	22	23
– 50–59 years	16	16
– 60+ years	7	6

F = female, M = male

The ratio of new referrals to follow-up appointments via telephone consultation was 1:2.73 (37:101) in the first survey's time period and 1:2.1 (58:122) in the second, with no statistical significance exhibited between the two ratios (*p* = 0.127).

### Satisfaction scores and preference

An average score of 114.6 out of 147 (range, 49–147; standard deviation = 26.9) was obtained in the first survey; whilst the second survey returned a mean score of 128.5 out of 147 (range 79–142; standard deviation = 13.9). The mean Medical Interview Satisfaction Scale score was statistically significantly higher in the second survey than in the first (*p* = 0.026). The subscale scores for both surveys are summarised in Table 3.

**Table 3.** Summary of MISS-21 mean scores

MISS-21 subscale (total score)	1st survey mean score (range)	2nd survey mean score (range)
Distress relief (42)	30.2 (6–42)	37.3 (21–42)
Communications comfort (28)	22.4 (7–28)	22.4 (14–28)
Rapport (56)	45.1 (11–56)	49.9 (29–55)
Compliance intent (21)	16.9 (9–21)	18.8 (9–21)
Total score (147)	114.6 (49–146)	128.5 (79–142)

MISS-21 = 21-point Medical Interview Satisfaction Scale

When comparing the subscale scores between the two time periods, statistical significance was achieved in the distress relief (*p* = 0.005), rapport (*p* = 0.041) and compliance intent (*p* = 0.021) subscales. The difference in communication comfort subscale scores did not reach statistical significance (*p* = 0.594) over the two surveys.

The average overall rating for telephone consultations in the first survey was 3.9 out of 5, with a significantly improved overall rating seen in the second survey, of 4.4 out of 5 (*p* = 0.039).

**Fig. 1.**
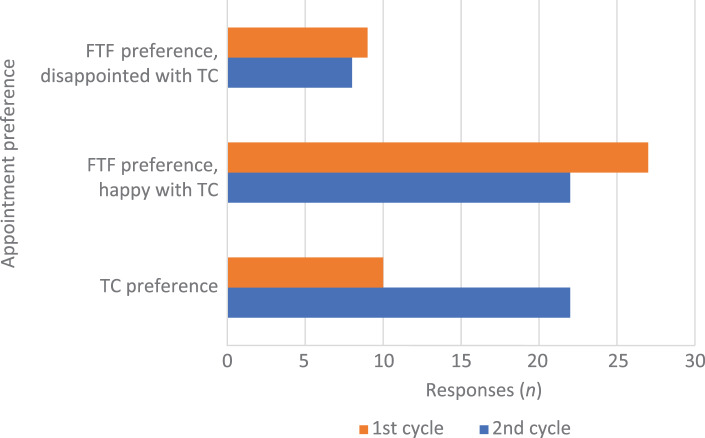
Patients’ preferences for telephone consultations versus face-to-face appointments, for both surveys. FTF = face-to-face appointment; TC = telephone consultation

Results of patients’ preferences for telephone consultations versus face-to-face appointments, for both surveys, are summarised in Figure 1. There was a significantly greater preference for telephone consultations over face-to-face appointments in the second survey when compared with the first survey (*p* = 0.03).

### Patients’ additional comments

Twenty-four additional comments were left in the first survey. Twelve (50 per cent) consisted of only positive feedback, with the remaining 12 entries offering constructive criticism. Four patients felt that the telephone medium resulted in limited consultation time, and consequently not all ENT-related issues were addressed. Three patients stated that their conditions required face-to-face assessment and treatment. The final four patient comments noted that symptoms were misunderstood and the patients were unsatisfied with the treatment provided.

Thirty-two additional comments were collected in the second survey, with 26 (81.3 per cent) consisting of only positive feedback and the remaining 6 comments (18.7 per cent) providing constructive criticism. Two comments described difficulties with communication due to landline issues, whilst two comments suggested longer telephone consultation times for better reviews. The final two patient comments noted that prescribed medication was not effective, preferring a face-to-face appointment for more appropriate management.

## Discussion

### Patient satisfaction and scoring

Assessing patient satisfaction serves as an important marker of clinical effectiveness whilst also serving as a predictor of treatment compliance.^4,9^ Patient satisfaction scores and health status frequently show a positive correlation.^10,11^ The Medical Interview Satisfaction Scale score, derived from a 29-question scoring system developed in the USA,^12^ was developed for the assessment of consultation satisfaction in the UK. Satisfactory internal reliability was demonstrated for this score, together with discrete overlapping aspects of satisfaction between the subscales.^4^ The Medical Interview Satisfaction Scale questionnaire was preferred over the Consultation Satisfaction Questionnaire for our project, as the latter had limited evidence of construct validity, together with a perceived time subscale that may be influenced by external factors. A similar study by Roberts and Partridge examining telephone consultations in respiratory clinics also utilised the Medical Interview Satisfaction Scale to good effect.^13^

Whilst the initial survey yielded overall positive results, some deficits were highlighted in the departmental meeting. The distress relief subscale (containing questions pertaining to patient's knowledge about their disease) had some concerning responses, with a quarter of patients submitting a mean score of less than 4 out of 7. Other areas, such as the rapport subscale (containing questions regarding the doctor–patient relationship established), showed more promising results. Of the patients, 58.3 per cent submitted a mean score of at least 6 out of 7, whilst 8.1 per cent submitted a mean score of less than 4 out of 7.

A current literature review including an in-depth analysis of three key papers^5–7^ was presented during the departmental meeting. Emphasis was placed on re-assessing patients’ understanding at the end of the conversation, and ensuring that their main concerns and expectations of the consultation had been addressed. A structured telephone consultation model was agreed upon (Table 1), comprising an amalgamation of Marshall and colleagues’ widely used ‘ISBAR’ (identify, situation, background, assessment and recommendation) tool for inter-professional communication,^8^ together with salient points adopted from telephone consultation guidance published in the *BMJ*.^5,6^

The second survey demonstrated significant improvement for almost all the subscales. Substantial improvement was seen in the distress relief subscale, with a mean score of 6.2 compared with a mean score of 5.0 in the initial survey. Only two respondents (3.8 per cent) submitted a mean score of less than 4 out of 7. The communication comfort subscale was the only section that did not show a statistically significant improvement (*p* = 0.594). Nevertheless, higher overall scores were seen in the second survey (mean score of 5.9 *vs* 5.63).

We believe that a number of factors were responsible for the improved patient satisfaction scores. We suggest that use of a structured model (Table 1) ensures an appropriate introduction to the telephone consultation. Such an introduction may be even more useful in the telephone consultation setting than in a face-to-face out-patient clinic, as patients may enter the former consultation in a completely different mind frame if also engaged in other activities. For example, we found that many patients took the call whilst at work and were consequently ill-prepared to discuss their condition.

Similarly, use of the model reminds the clinician to summarise the consultation, checking that patient concerns and expectations have been satisfactorily addressed. This was highlighted in our improved scores pertaining to the distress relief subscale.

Furthermore, discussion of possible shortcomings from the initial survey is likely to have raised clinician awareness of patients’ perceptions of telephone consultations, and heightened awareness of limitations of telephone consultations. The score improvements may also be associated with clinicians gaining a further two months’ experience in telephone consultations by the start of the second survey. Additionally, some patients may have become accustomed to telephone consultations in the second survey's timeframe, now widely employed in out-patient clinics across specialties and in primary care.

### Telephone consultation preference

Whilst face-to-face appointments are considered the norm, remote telephone consultations have been widely used as an acceptable substitute during the Covid-19 pandemic.^14^ Prior to the outbreak, a quarter of healthcare staff and patient interactions occurred over the telephone, in both the USA and UK.^6^ Medical hotlines had been set up to tackle out-of-hours services, serving as a triaging service for either urgent emergency hospital review or scheduled community appointments.^15^ Secondary and tertiary care clinics had also started to integrate telephone consultation services,^16–19^ as demonstrated by specialist nurse-led post-natal care and prostate cancer follow-up clinics.^17,19^ Additionally, a respiratory medicine clinic demonstrated similar 21-point Medical Interview Satisfaction Scale satisfaction scores from both telephone and traditional face-to-face consultations, citing the lack of travelling and waiting time as major advantages for the participating patients.^13^

Telephone consultations have proven to be acceptable and sometimes desirable alternatives to out-patient visits, with positive satisfaction scores and safe levels of care maintained.^17^ Shorter lists of patient reviews (allowing more appointments for new referrals), a decrease in clinician workload and a reduction in non-attendance were quoted as the main positives for clinicians.^20^

The Covid-19 pandemic has resulted in an even greater push towards remote consultations. Subramanian *et al*. described a new voice response system implemented by tertiary mental health services in India,^21^ whilst Calton *et al*. denoted increased remote consultation use in their palliative medicine clinics.^2^

Our quality improvement project found a substantial increase in telephone consultation preference in the second survey, together with an improvement in patient satisfaction. Five comments cited similar advantages for remote consultations: no need to take time off work, no transport issues and less waiting time in clinics.

### Looking ahead and beyond the pandemic

Although the literature has shown high patient satisfaction results associated with telephone consultations, many healthcare professionals and patients remain ambivalent about telephone calls. Whilst valuing the aforementioned advantages, the inability to examine and visualise signs to help support a diagnosis is a significant drawback.^6,14^ Remote video consultations offer a solution to address this drawback to a limited degree, although additional equipment and training for clinicians and patients is required.^1^

The significant improvement in patient satisfaction scores is promising and gives us the confidence of going forwards with telephone consultations, even after the Covid-19 pandemic. Telephone consultations may play a large role in the national response to the expected backup in demand for elective activity following the resolution of the pandemic. Telehealth may help lessen the impact of out-patient up-demand on healthcare workers and subsequently reduce the forecasted gap in service provision.^21,22^

### Limitations

The main limitation was the lack of data collected containing any identifiable patient data and information regarding the disease being followed up. Furthermore, patients with limited internet access would not have been able to fill in the questionnaire, which is reflected in our questionnaire completion rate (34.8 per cent and 29 per cent for the first and second survey respectively). This is notably lower when compared to a similar study by Meakin and Weinman (72.6 per cent response rate), thus potentially creating a selection bias in our project.^4^ Finally, we did not record the length of time spent per telephone consultation; however, the number of negative comments regarding lack of consultation time decreased from four to two.

• The coronavirus disease 2019 pandemic has allowed the fruition of telehealth services in out-patient settings
• A structured approach to telephone consultations had a positive impact on patient satisfaction
• Patient satisfaction scores showed a significant increase in telephone consultation preference over traditional appointments
• Advantages for patients include: decreased waiting time, less disruption to working hours and elimination of travelling obstacles
• Advantages for clinicians include: more appointment slots for new referrals, decreased physician workload and reduced non-attendance

## Conclusion

Whilst the Covid-19 pandemic has created countless challenges for health services, a window of opportunity has presented itself for the expansion of remote consultation services. Whilst still in its infancy in our department, we have shown significant improvements in terms of patient satisfaction following telephone consultation. In addition, there was an increase in patients’ preference for telephone consultations over face-to-face appointments following clinician education and the implementation of a structured telephone consultation model.

## Competing interests

None declared.

## Appendix 1. Telephone consultation feedback questionnaire

1. Please select your age group:
  ▫ Under 18  ▫ 18–29  ▫ 30–49  ▫ 50–59  ▫ 60+
2. Gender:
  ▫ Male  ▫ Female  ▫ Prefer not to say
  Medical Interview Satisfaction Scale 21 (Q3–Q23)
  Please rate the following, from 1 (very strongly disagree) to 7 (very strongly agree)
3. The healthcare professional told me what my medical problem/s were.
4. After the telephone consultation, I know just how serious my illness is.
5. I was told all I wanted to know about my illness.
6. I am not really certain about how to follow the advice given.
7. After talking with the healthcare professional, I have a good idea of how long it will be before I am well again.
8. The healthcare professional seemed interested in me as a person.
9. The healthcare professional seemed warm and friendly to me.
10. The healthcare professional seemed to take my problems seriously.
11. I felt embarrassed whilst talking.
12. I felt free to talk about private matters with the healthcare professional.
13. I was given a chance to say what was really on my mind.
14. I really felt understood during the consultation.
15. I was not allowed to say everything I wanted to about my problems.
16. The healthcare professional did not understand my main reason for coming.
17. This is a healthcare professional I would trust with my life.
18. The practitioner seemed to know what (s)he was doing.
19. The practitioner has relieved my worries about my illness.
20. The practitioner seemed to know just what to do for my problem.
21. I expect that it will be easy to follow the advice given.
22. It may be difficult for me to do exactly what the practitioner told me to do.
23. I'm not sure the advised treatment will be worth the trouble it will take.
24. How did the telephone consult compare to a face-to-face clinic appointment?
25. Overall satisfaction of telephone conversation:
26. Any additional comments welcome ___________________________________
  _______________________________________________________________________
